# Notes from Private Practice

**Published:** 1877-11

**Authors:** H. Banga

**Affiliations:** Chicago


### Clinical Reports.

### NOTES FROM PRIVATE PRACTICE.

*Two Cases of Stricture of the Urethra.*

As there is still a great difference of opinions among surgeons respecting the curability of organic stricture of long standing, I desire to record the favorable result obtained by gradual dilatation in an organic stricture of twenty years' duration. Abernethey and others advise to begin the exploration of a strictured urethra with fine bougies "corresponding to the stream;" while Dittel sees in the early use of larger bougies a safer method, because the fine instruments are too liable to make false passages.

*Case I.*—I. B., aged 43 years, a sailor, had gonorrhœa twenty-two years ago, and has been suffering from stricture of the urethra for twenty years. When he came under my care, in February, the stream of his urine was of the size of a knitting needle; the urine was turbid and alkaline; micturition frequent. He complained of pain in the sides, costiveness and general weakness. The stricture occupied the membranous portion of the urethra where a hard tumor could be felt through the perineum. I succeeded in passing a No. 1 French bougie through the stricture; but twice subsequently I failed with the same instrument, and on the third trial, when it seemed to pass through, I convinced myself it was pursuing a false passage. I therefore desisted from all further attempts until the damage could be repaired.

I then introduced No. 26 of a set of metallic sounds (the largest size which would be admitted by the external orifice of the urethra) as far as the stricture, and retained it there for twenty minutes. This being repeated eight days in succession, the lower part of the stricture was stretched to such an extent that on trying a small bougie it passed through without difficulty, and afterward the stricture dilated gradually, though slowly, so that at the end of three months the No. 26 could be admitted. By this time the urine had become clear and the patient had greatly improved in health. Three months later I could ascertain that the caliber of the urethra had not decreased, yet admitting the No. 26 as easily as before.

Some practitioners would, perhaps, have shortened the treatment of this case by internal urethrotomy. But if the patient can only occasionally be seen, I do not think it prudent to perform an operation which possibly might jeopardize life, to say nothing of the reputation of the physician.

*Case II.*—This case is remarkable in its anatomical features, since there were all the symptoms of stricture, obstruction to the bougie alone excepted. Others might therefore not call it a stricture; but I treated the difficulty as such, and succeeded in relieving it. The brief history of the case is this: A young man of 33 called on me in June last, on account of several disorders, following an attack of gonorrhœa, which he had three years ago. His complaint was a steady diminution of the stream and a change in its direction, frequent micturition, turbidity of the urine, intercurrent pains in both sides, habitual costiveness, and a general depression of the system, which the patient attributed to the medicines he had been taking for nearly three years. From this history, I concluded he had stricture and introduced a metallic bougie, No. 26, but to my great surprise it passed along the urethral channel without meeting any obstruction. The external examination of the urethra, through the rectum, failed to detect any change in texture or sensibility. Under these circumstances, the only way of explaining the symptoms was to suppose that granulations, springing from some ulcerated patch in the urethra, and soft enough to give way to the catheter caused the diminution of the stream, and, as a consequence, the incomplete evacuation of the bladder. The only objection I could find to this supposition was, that there was no trace of blood on the instrument. I introduced, twice a week, a metallic bougie, No. 26, coated with an ointment containing tannic acid, and left it in situ from thirty to sixty minutes. The stream soon grew larger and continued so after each introduction of the instrument, so that, after six weeks, the patient was discharged completely cured. I should not be surprised if after some months the patient should return, complaining of the same disorder, because I think that this was the first stage of stricture, and that I must expect the second, namely, retraction of tissue. However, I learn from the patient now, two months after his discharge, that he is feeling quite well, and notices no diminution in the stream of urine.

Dr. H. Banga.

Chicago, 20th of September, 1877.

